# Inbreeding, Allee effects and stochasticity might be sufficient to account for Neanderthal extinction

**DOI:** 10.1371/journal.pone.0225117

**Published:** 2019-11-27

**Authors:** Krist Vaesen, Fulco Scherjon, Lia Hemerik, Alexander Verpoorte

**Affiliations:** 1 School of Innovation Sciences, Eindhoven University of Technology, Eindhoven, The Netherlands; 2 Human Origins Group, Faculty of Archaeology, University of Leiden, Leiden, The Netherlands; 3 Biometris, Mathematical and Statistical Methods, Wageningen University, Wageningen, The Netherlands; Universitat de Barcelona, SPAIN

## Abstract

The replacement of Neanderthals by Anatomically Modern Humans has typically been attributed to environmental pressure or a superiority of modern humans with respect to competition for resources. Here we present two independent models that suggest that no such heatedly debated factors might be needed to account for the demise of Neanderthals. Starting from the observation that Neanderthal populations already were small before the arrival of modern humans, the models implement three factors that conservation biology identifies as critical for a small population’s persistence, namely inbreeding, Allee effects and stochasticity. Our results indicate that the disappearance of Neanderthals might have resided in the smallness of their population(s) alone: even if they had been identical to modern humans in their cognitive, social and cultural traits, and even in the absence of inter-specific competition, Neanderthals faced a considerable risk of extinction. Furthermore, we suggest that if modern humans contributed to the demise of Neanderthals, that contribution might have had nothing to do with resource competition, but rather with how the incoming populations geographically restructured the resident populations, in a way that reinforced Allee effects, and the effects of inbreeding and stochasticity.

## Introduction

A long-standing enigma in palaeoanthropology is the demise of Neanderthals approximately 40 kya [1]. There is general agreement that―after a long period of largely separated coexistence [2–6]―their disappearance roughly coincides with migration events starting around ~60 kya by Anatomically Modern Humans (AMHs) from Africa into the Near East and Europe and that, accordingly, AMHs took over the territories previously occupied by our sister species [7–12]. What is uncertain however, are the causes of Neanderthal extinction. It has been attributed to a wide variety of intensely debated factors, including climatic change ([13–17], but see [18–19]), epidemics [20–21], a superiority of AMHs over Neanderthals in competing for the same resources ([22–34] but see [18]). Models have, not surprisingly, confirmed that if such superiority is assumed [35–41], Neanderthals would indeed have been likely to go extinct.

Here we argue that no such contested factors might be needed to account for the demise of Neanderthals. We present two independent models that capture the internal dynamics of Neanderthal populations―the models thus ignore, among other things, competitive interactions with AMHs―and that suggest that the disappearance of Neanderthals might have resided in the small size of their population(s) alone. Accordingly, our study substantiates the suggestion, made in passing by French [42], that “it may simply be the case that Neanderthal populations declined below their minimum viable population threshold”.

Genetic studies, indeed, indicate that Neanderthal effective population size―the size of the ideal population that would undergo the same amount of random genetic drift as the actual population [43]―was already small by ~400 kya, amounting to no more than 3,000–3,900 individuals, a level that was sustained almost up till the species’ extinction ~38 kya [44–46]. Rogers et al. [47] report a higher number for the effective size of the global Neanderthal population, but also show that this global population was subdivided in very small and highly isolated local populations (each, so to speak, with its own, small effective size). Likewise, Castellano et al. [48] conclude, based on a comparison between the genome of three Neanderthal individuals and the genome of present-day modern humans, that Neanderthal genetic diversity was limited and that Neanderthal populations were small and isolated from one another. The archaeological data point in the same direction ([49], and references therein); and Bocquet-Appel and Degioanni [50] estimate that the Neanderthal census metapopulation was in the range of a mere 5,000–70,000 individuals.

The models presented here implement three basic factors that, according to conservation biology (the field from which our models were drawn), would put such small populations at risk of extinction: inbreeding, Allee effects and stochasticity ([51]; in archaeology, see Finlayson [52]). Inbreeding depression refers to the reduction in fitness of individuals that arise from matings between genetic relatives, matings thus that are more likely to occur in small populations. Inbreeding, which seems to have been common in Neanderthals [44, 53–55], might lead to a lower fitness because it increases the chances of the expression of recessive, deleterious traits and because homozygotes often have a general disadvantage relative to heterozygotes. Harris and Nielsen [56] estimate that, due to inbreeding, Neanderthals had at least 40% lower fitness than modern humans on average. Allee effects refer to the effects that population density has on reproduction and, thus, on population growth [51]. At lower densities, the case we are concerned with here, growth rates might drop due to problems in mate-finding, and to several problems that highly cooperative species, such as Neanderthals, are particularly susceptible to, including low availability of helpers in cooperative hunting, defending kills from kleptoparasites, and allo-parenting [57]. Finally, stochastic, annual fluctuations in births, deaths and sex ratio are more likely to place smaller populations on a trajectory towards extinction than bigger ones [51].

Our models indicate that these factors alone could have resulted in Neanderthal extinction, even if Neanderthals and AMHs were identical in terms of individual-level traits that are deemed relevant to persistence or extinction (e.g., cognitive and technological ability, sociality).

## Materials and methods

The specific question our study aims to address is whether inbreeding, Allee effects and stochasticity are sufficient to explain the disappearance of Neanderthals. To that end, we develop two separate models, both of which track Neanderthal population growth over time. Crucially, since we want to avoid making assumptions about the superiority of AMHs over Neanderthals, both models are parameterized based on estimates for AMHs, estimates that pertain to, among other things, reproduction, mortality, and inbreeding (see below, S1 Appendix, and S6 and S7 Tables).

The first model is a deterministic matrix model, the second an individual-based stochastic simulation model (IBM). The matrix model served two purposes: to calibrate the IBM and to validate some of the results of the IBM. Since, in contrast to the matrix model, the IBM allowed us straightforwardly to introduce inbreeding and stochasticity, it was our primary resource for obtaining the results reported here. More specifically, we used the IBM to determine, separately, the levels of inbreeding and the levels of Allee effects that put Neanderthal populations at risk of extinction; and to determine Neanderthal’s vulnerability to extinction in a scenario involving both inbreeding and Allee effects. Subsequently, we set these results against what―as it comes to Neanderthal population size, inbreeding and Allee effects―can be inferred from the literature.

Below we provide a general, non-technical overview of the research set-up. For a full description, please consult S1 Appendix.

### General description of the basic models

The deterministic model consists of a Leslie matrix, a type of matrix that is commonly used in conservation biology to model population growth [58]. The matrix summarizes, for each of a population’s age classes, yearly survival and reproduction. Our model assumes a sex ratio of 1:1 and, because they are the limiting factor for reproduction, only considers females. Accordingly, the first row of the matrix contains, for each of the age classes, the average number of female offspring per female per year. The subdiagonals represent the yearly survivals of females; these values are on the subdiagonals because, whenever a female survives in a given year, she will move to the next age class. The growth factor of the population―the factor by which the population will annually be multiplied―is given by the dominant eigenvalue of the matrix. If this factor is less than 1, the population will have a negative growth rate and goes extinct.

For the IBM, we relied on VORTEX [59–61], a software package used by conservation biologists to perform Population Viability Analyses of endangered wildlife species. VORTEX simulates the annual life events (e.g., sex determination, breeding, mortality) that might occur to each of the individuals within a given population, and records over time, in discrete time steps, the characteristics of these individuals as well as those of the population as a whole. Occurrences of events are probabilistic. Demographic stochasticity (relating to annual fluctuations in, e.g., sex ratios, births and deaths) is thus inherent to VORTEX models.

### Calibration

We calibrated the two models by checking whether, under similar parameter settings, they were able to produce similar trends in Neanderthal population growth. At this stage, we didn’t include inbreeding or Allee effects.

The primary input parameters for both models were derived from estimates of female reproduction in extant hunter-gatherers [62] and from the West model life table, Level 5 [63]. In general, such model life tables summarize the mortality events of an ideal population. West tables are the most commonly used in human palaeo-demography; Level 5 is closely matched by the mortality profiles of extant hunter-gatherers [64].

The parameters used in the matrix model are shown in S1 Appendix, S1 Table. Calculating the dominant eigenvalue of the corresponding matrix yields a population growth factor of 1.008, which corresponds to a relative growth rate of 0.80%.

The parameters used in the VORTEX model are summarized in S1 Appendix, S5 and S6 Tables. For various initial population sizes (*N*_*0*_ = 50; 100; 500; 1,000; 5,000), we ran ten simulation runs, each simulating a time span of 100 years. The relative growth rate of these simulations was, on average, 0.76%. This value is slightly lower than the relative growth rate obtained from the matrix model. However, it has been theoretically established that, in general, growth rates of stochastic models are less than the growth rates of deterministic models [65].

### Inbreeding and stochasticity

In conservation biology there is no standard procedure for implementing inbreeding in deterministic matrix models. Therefore, we relied on our IBM to model inbreeding. In VORTEX, inbreeding depression is modeled in terms of its effects on infant survival. It is governed by two parameters: the number of lethal equivalents, *I*, which is the number of recessive alleles carried in a heterozygous genome that would be lethal if carried in the homozygous state; and the percentage, *f*_*i*_, of the inbreeding depression caused by such lethal alleles rather than by other genetic mechanisms (e.g., a general disadvantage of homozygotes). Based on observations of several species, VORTEX sets *f*_*i*_ at 50% by default.

For various initial population sizes (*N*_*0*_ = 50; 100; 500; 1,000; 5,000) and various value of *f*_*i*_ (viz., *f*_*i*_ = 30;50;70), and again starting from West table Level 5, we determined, by simulating widely over *I*, the parameter *I*_*risk*_. *I*_*risk*_ is the lowest value of *I* that yields at least one extinction event in ten simulation runs, each run simulating over a time span of 10,000 years. Furthermore, we determined, *I*_*sure*_, which is the lowest value of *I* that yields extinction in all of the ten runs. Note that VORTEX models that incorporate inbreeding generally run very slow, and do so especially when carrying capacity is high. Therefore we set *K* at 5,000, unlike we did in the basic model (where carrying capacity was set at *K* = 10,000). This doesn’t affect our conclusions (see below).

### Allee effects, with and without stochasticity

The dependence of female breeding rates on population size is commonly (including in VORTEX) captured by an equation that, when simplified to cover the most conservative scenario, tells us that the fraction of females breeding at population size *N*, *E*, is given by

$$E=\frac{p_{0}}{100}\frac{N}{N+A},$$ (1)

where *p*_0_ is the percentage of females breeding in the absence of population size constraints, and *A* is a parameter describing the strength of the Allee effect (see equations S4 and S5 in the S1 Appendix).

Accordingly, in the IBM we simulated widely over *A*, and determined, for various initial population sizes (*N*_*0*_ = 50; 100; 500; 1,000; 5,000), *A*_*risk*_, which is the lowest value of *A* that leads, in ten simulation runs (each run comprises 10,000 years), to at least one extinction event. Also, we determined *A*_*sure*_, which is the lowest value of *A* for which all ten runs result in extinction.

In order to validate our results, we introduced the Allee equation used in the IBM into the matrix model, and set the results against the results obtained from the IBM.

### Inbreeding, Allee effects and stochasticity

Estimates of *I* for modern humans [66–70] range from 0.58 [69] to 2.2 [66]. Gao et al. [69] point out that, since these estimates are based on reported deaths after birth and thus do not take into account prenatal deaths, the actual number of lethal equivalents might be higher; the authors surmise that prenatal deaths might increase *I* by one additional lethal equivalent (resulting in a maximum value of *I* = 3.2).

To assess the combined effects of inbreeding, Allee effects and stochasticity, we ran simulations for the highest *I*-value just reported (i.e., *I* = 3.2). More specifically, by widely varying over *A*, we determined for this *I*-value, and for various initial population sizes (*N*_*0*_ = 50; 100; 500), *A*_*risk*_.

Although *I* = 3.2 is the least conservative value among the values found in the literature, our choice was motivated by the results obtained in scenarios that involved inbreeding alone (i.e., the results suggested that even at *I* = 3.2 the impact of inbreeding would be relatively small; see Results), and by the large computational demands of VORTEX in scenarios that combine Allee effects, inbreeding and very lengthy timespans (10,000 years). Also due to resource constraints, we did not determine *A*_*sure*_, restricted *N* to the range 0–500, and set carrying capacity at *K* = 5,000.

## Results

### Inbreeding and stochasticity

**Fig 1:**
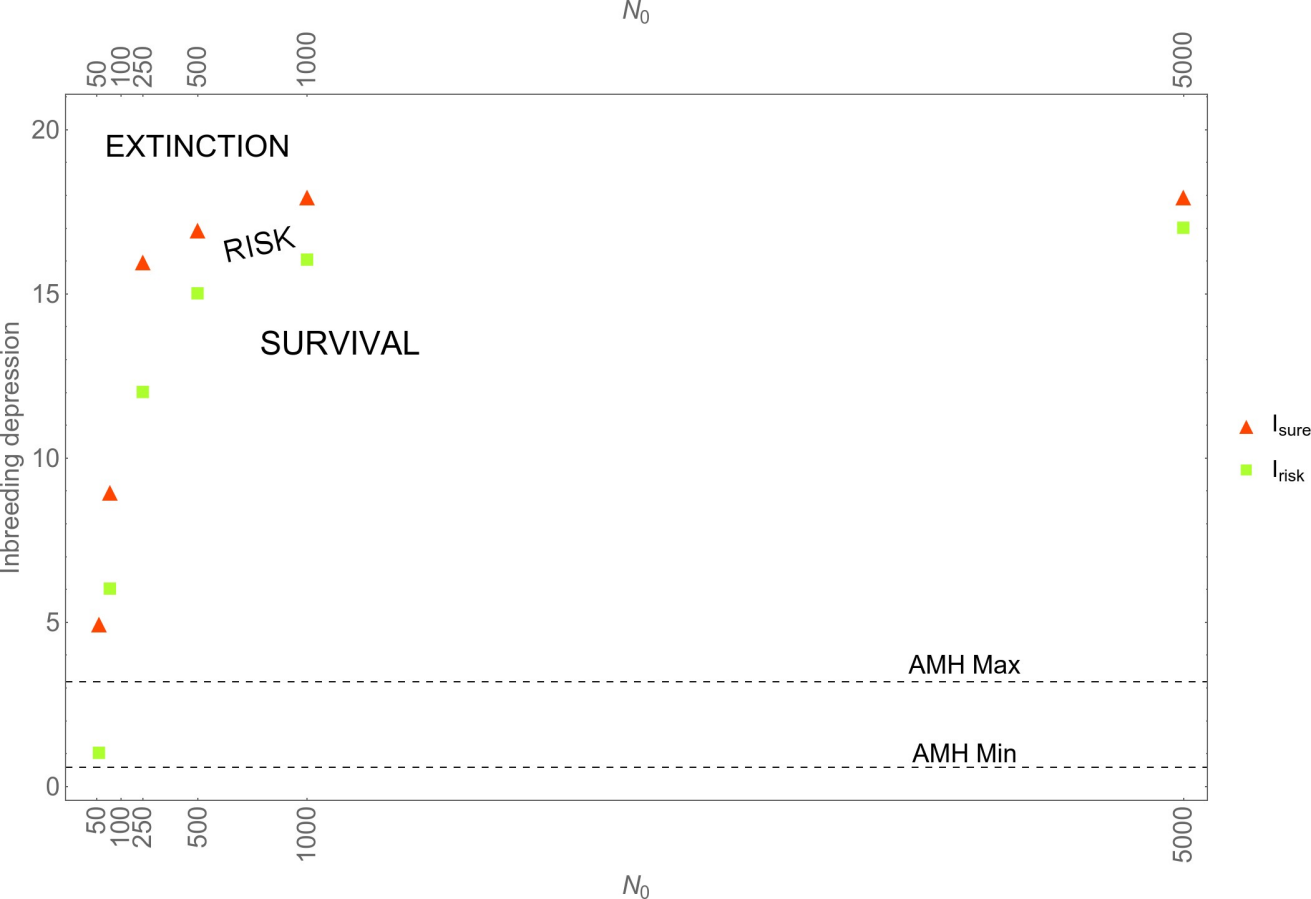
Inbreeding and stochasticity. *I*_*risk*_ (lowest value of inbreeding depression parameter *I* that results in at least one extinction event in ten simulation runs) and *I*_*sure*_ (lowest value of inbreeding depression that results in extinction in all simulation runs) for various initial population sizes *N*_*0*_ and *f*_*i*_ = 50% (for *f*_*i*_ = 30;70, see S1 Appendix). The horizontal dotted lines mark the range of values of *I* observed in AMHs.

Fig 1 plots *I*_*risk*_ and *I*_*sure*_ for initial populations of size *N*_*0*_ = 50; 100; 500; 1,000; 5,000, assuming *f*_*i*_ = 50. Fig 1 comprises three regimes: below the lower, green squares populations can be expected to survive (SURVIVAL); above the upper, red triangles populations die out (EXTINCTION); populations in between run a risk of extinction (RISK). The latter regime is where stochastic effects occur, with higher risks of extinction closer to the upper, red triangles.

In virtually all of the scenarios, the available estimates for modern humans (ranging from 0.58 to 3.2; see above) fall in the regime of survival. Only when *N*_*0*_ *= 50*, Neanderthals face a risk of extinction, i.e., *I*_*risk*_ falls in the range 0.58–3.2 (the same holds when *f*_*i*_ = 30; 70, see S4 Fig). Given that the lowest Neanderthal census size reported in the literature is 5,000 [50], and on the assumption that Neanderthals carried the same average number of lethal equivalents as AMHs, it is unlikely that Neanderthals would have disappeared due to inbreeding alone. Even if the meta-population comprised hundred isolated bands of 50 individuals each, e.g., because these bands were interspersed by AMH bands (see Discussion), it is unlikely that inbreeding alone would have resulted in extinction. Recall that *I*_*risk*_ is the lowest value of *I* that yields at least one extinction event in ten simulation runs, each run simulating over a time span of 10,000 years. Accordingly, if inbreeding were the primary cause of Neanderthals extinction, one would need to make the extraordinary assumption that each of the hundred bands experienced an extinction event that had only a one out of ten chance of occurring.

In the simulations, *K* was set at *K* = 5,000. But our conclusions would also hold if, as we did in the basic model, *K* had been set at *K* = 10,000. Carrying capacity exerts a downward push on populations that are approaching it. Increasing *K* thus would make it even more unlikely that inbreeding alone would have yielded extinction in populations that did not go extinct at lower *K*.

### Allee effects, with and without stochasticity

**Fig 2:**
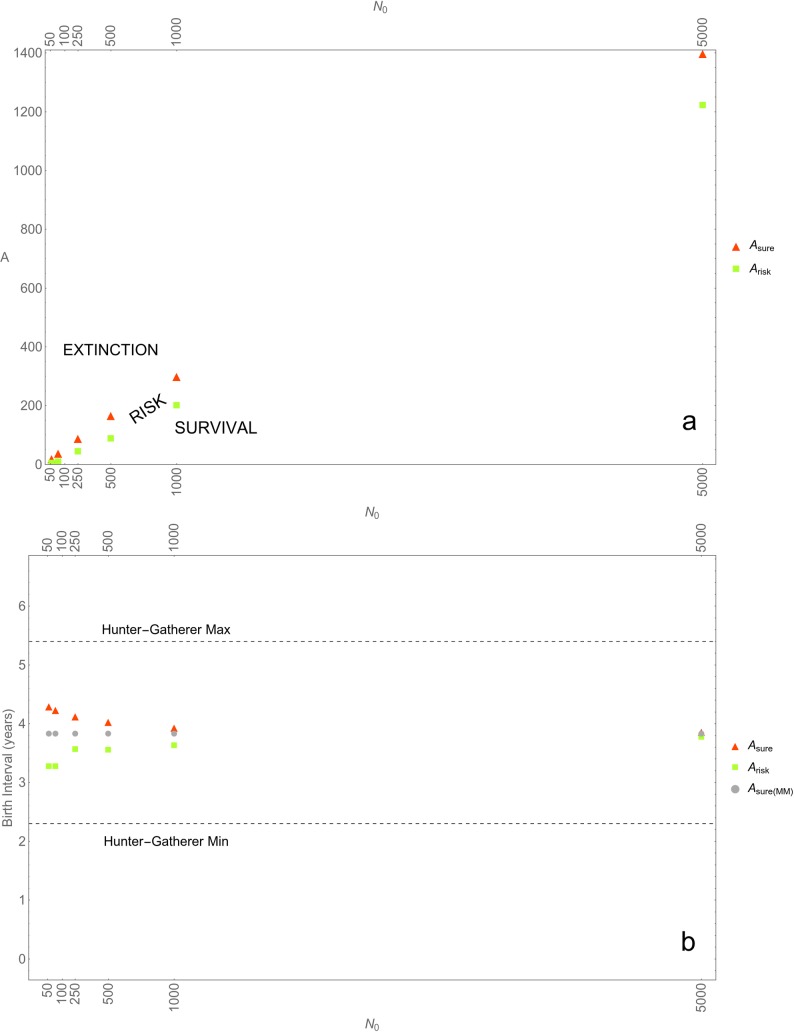
Allee effects, with and without stochasticity. (a) *A*_*risk*_ (lowest value of Allee parameter *A* that results in at least one extinction event in ten simulation runs) and *A*_*sure*_ (lowest value of Allee parameter *A* that results in extinction in all simulation runs) for various initial population sizes *N*_*0*_. **(b)** Birth intervals corresponding to *A*_*risk*_ and *A*_*sure*_, as well as birth intervals corresponding to the *A*_*sure*_ values obtained in the non-stochastic matrix model (labeled *A*_*sure(MM)*_). The horizontal dotted lines mark the range of values of birth intervals observed in contemporary hunter-gatherers.

Fig 2A represents *A*_*risk*_ and *A*_*sure*_ for populations of size *N*_*0*_ = 50; 100; 500; 1,000; 5,000. Again, Fig 2A comprises three regimes (viz., SURVIVAL, RISK and EXTINCTION).

For most animal species, including AMHs and Neanderthals, estimates of *A* are unavailable [71]. Yet, Eq (1) allows us indirectly to assess Allee effects. In all of our simulations, the percentage of females breeding *without* Allee effects, *p*_*0*_, was set at 0.33, which corresponds to a birth interval of 3 years, which in turn corresponds to the average birth interval reported by Kelly [62] among extant hunter-gatherers and with the birth interval estimated for Neanderthals by Lalueza-Fox and colleagues [72]. Consider now a population of *N =* 1,000, for which *A*_*sure*_ equals 301 (see Fig 2A). Eq (1) tells us that Allee effects need to reduce the percentage of females breeding to 0.25 for the population to become extinct. This percentage corresponds to a birth interval of 4 years, a value that falls well within the range of birth intervals reported by Kelly [62] (viz. 2.3–5.4). In other words, for extinction to occur, it is enough that Allee effects produce birth intervals that are common among extant hunter-gatherers. Performing the same calculation for all *N*’s results in Fig 2B. It appears that all of the birth intervals neatly fall in the range observed among extant hunter-gatherers.

The regime in between the red and green dots illustrates the effects of stochasticity. In this regime, fluctuations in births, deaths and sex ratio determine whether or not, in a time span of 10,000 years, an actual extinction event takes place. So even at *A*_*risk*_, when Allee effects are relatively small (e.g., for *N =* 1,000, *A*_*risk*_ is 200, which corresponds to a birth interval of 3.6 years), random events might lead to extinction.

Fig 2B also plots the birth intervals inferred from the results of the matrix model (grey dots). The values, again, fall within Kelly’s range, as does the value for a population of *N* = 70,000 (not depicted in Fig 2B; birth interval of 3.84 years). Importantly, the results of the matrix model and of the IBM fall within the same range; and at higher population sizes, where stochastic effects are expected to be small, the values of *A*_*sure*_ obtained in the matrix model and in the IBM start to converge.

Note that in our models, Allee effects only comprise the effects that population size has on the percentage of females breeding. Our models thus exclude the effects that low population numbers might have on survival rates (e.g., lower infant survival due to shortages in allo-parents or cooperative hunts), and therefore likely *underestimate* the challenges faced by small populations. In sum, Allee effects probably were a key, and perhaps even a sufficient, factor in the demise of Neanderthals.

### Inbreeding, Allee effects and stochasticity

**Fig 3:**
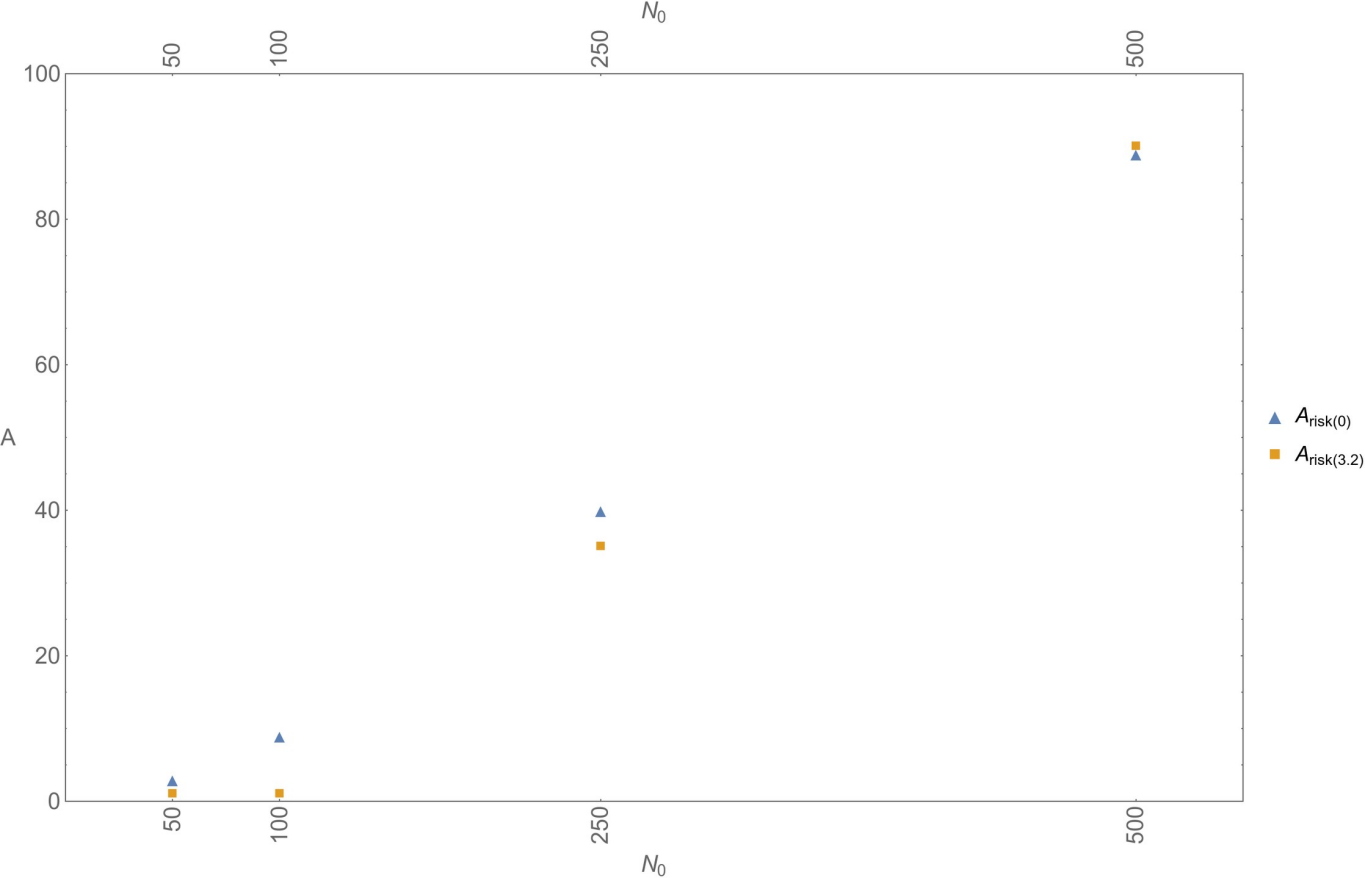
Inbreeding, Allee effects, and stochasticity. *A*_*risk*_ (lowest value of Allee parameter *A* that results in at least one extinction event in ten simulation runs) for a scenario without inbreeding (blue triangles; *A*_*risk(0)*_) and a scenario with inbreeding (orange squares; *A*_*risk(3*.*2)*_) (inbreeding depression parameter at *I* = 3.2).

Fig 3 plots, for various initial population sizes *N*_*0*_, *A*_*risk(0*.*0)*_ and *A*_*risk(3*.*2)*_, which denote, respectively, *A*_*risk*_ obtained in a scenario with only Allee effects, and *A*_*risk*_ obtained in a scenario with Allee effects and inbreeding—the latter set at the highest *I*-value reported in the literature (viz. *I* = 3.2). It appears that even at this highest *I*-value inbreeding leads to a reduction in *A*_*risk*_ only at lower *N*_*0*_; already at *N*_*0*_ = 500, the values of *A*_*risk(0*.*0)*_ and *A*_*risk(3*.*2)*_ converge. In order to check whether at lower *I*-values inbreeding would still have an effect on *A*_*risk*_ we performed some extra simulations, now setting *I* at *I* = 0.58; 2.2, and *N*_*0*_ at *N*_*0*_ = 50; 100. It turned out that such lower levels of inbreeding reduce *A*_*risk*_ when *N*_*0*_ = 50, but not when *N*_*0*_ = 100.

These findings are in line with the observation above that, if Neanderthals carried the same number of lethal equivalents as AMHs, inbreeding would have had an effect only at the sub-population level. In such a scenario, inbreeding would occasionally—i.e., when Neanderthals had the stochastic odds against them—lead to a local extinction event, thereby accelerating a process of decline that was primarily driven by Allee effects.

## Conclusion and discussion

Our results support the hypothesis that the disappearance of Neanderthals might have been the result of a demographic factors alone, that is, the result merely of the internal dynamics that operate in small populations. Our conclusions are consistent with but go beyond the conclusions of a recent study by Kolodny and Feldman [73]. Based on a series of mathematical models, these authors too argue that no external factors (climate, epidemics) nor a superiority of AMHs in resource competition are needed to account for Neanderthal extinction. Their models suggest that migratory dynamics―with more migration happening from Africa into Europe by AMHs than migration from Europe into Africa by Neanderthals―might have been sufficient to result in the replacement of Neanderthals by AMHs. While Kolodny and Feldman’s models indeed do not assume a competitive advantage for either species (but see S1 Appendix), they do take for granted that Neanderthals and AMHs competed for the same habitats. Our study shows that even without this contested assumption ([74]; see also the literature on competition avoidance among extant hunter-gatherers, e.g., [75] and references therein), Neanderthal extinction might have taken place.

If Neanderthals lived in small populations since ~400 kya [44–46], why did it take so long for them to become extinct? A first relevant consideration concerns *demographic* stochasticity. We have seen that annual fluctuations in births, deaths and sex ratio might determine whether and when a small population disappears. So our results are consistent with a scenario in which a small population of Neanderthals persists for several thousands of years, and then, due to a stroke of bad luck, disappears. Furthermore, for the sake of simplicity, our models do not take into account *environmental* stochasticity. That is, the models work with fixed probabilities for mortality, fertility and sex ratios―fluctuations are thus simply caused by probabilistic sampling (demographic stochasticity). In natural conditions, though, the probabilities themselves will vary, according to random fluctuations in the environment (environmental stochasticity). Note that these fluctuations do not correspond to the millennial trends observed in the palaeoclimatic record, but occur at much lower temporal scales (e.g., a couple of years of drought, an epidemic among prey). They should be understood as natural variations around a mean, rather than as external forcings, such as the ones that some scholars have claimed to be responsible for the demise of Neanderthals (e.g., climatic change [13–16] or volcanic eruptions [17]). Importantly, in a given year, demographic and environmental stochasticity might very well work in opposite directions; environmental conditions, for instance, might be favorable and alleviate the stress induced by demographic stochasticity. In fact, it will very rarely happen that a metapopulation comprising several sub-populations has *all* the stochastic odds against it, that is, it will only very rarely happen that, for a significant amount of time, environmental variability produces low fertility rates *and* high death rates, and additionally, demographic stochasticity produces low fertility rates *and* high death rates *and* unfavorable sex ratios, and this in all of the metapopulation’s sub-populations. But in the very long run, such an unfavorable scenario eventually will take place. Accordingly, it is not implausible that, despite regular local extinction events [76], a small metapopulation manages to survive over prolonged stretches of time but eventually dies out due to its overall size and stochasticity. Noteworthy, there is nothing unusual about the persistently small size of Neanderthal populations. Hominin populations likely were small throughout the Pleistocene [77].

We suggest that AMHs might still have contributed to the extinction, but not necessarily by engaging in competition with or outcompeting Neanderthals. The mere interspersal of AMH sub-populations between Neanderthal sub-populations reduced the opportunities for intra-breeding and migratory activity among the latter. The resulting small and isolated sub-populations (as documented by [47–49,76,78]) would be increasingly vulnerable precisely to the factors examined in the current paper (viz. inbreeding, Allee effects and stochasticity), and thus to extinction. As such, the presence of modern humans in Eurasia would have accelerated a process that, at some point, was likely to have occurred anyway. Stated otherwise, the arrival of AMHs would have been a contributory factor rather than the cause of the extinction. Importantly, population-level characteristics―e.g., many of the characteristics that conservation biology has shown to be critical for a species’ persistence, including population size, species distribution, *intra*specific variability, and patterns of dispersal―might also account for the successful range expansion of AMHs. In other words, our species’ success need not be the result of a superiority in its individual-level traits.

An explanation solely in terms of the internal dynamics of the Neanderthal population, as the one presented here, serves as a null hypothesis against which competing, and less parsimonious, hypotheses are to be assessed. Regardless of whether external factors (climate or epidemics) or factors related to resource competition played a role in the actual demise of Neanderthals, our study suggests that any plausible explanation of the demise also needs to incorporate demographic factors as key variables.

## Supporting information

S1 Appendix Supplementary information. (DOCX)

S1 Table Yearly survival probabilities based on the life tables published by [63]. (DOCX)

S2 Table Sensitivities and elasticities in the density-independent model (see third and fourth column). (DOCX)

S3 Table Sensitivities and elasticities in the density-dependent model (see third and fourth column). Note that the Allee effect is included with specific parameter values. (DOCX)

S4 Table *A*_*sure(MM)*_ and corresponding birth interval, for various initial population sizes *N*_*0*_ (see Fig 2B in main text). (DOCX)

S5 Table Comparison of 5y-to-1y conversion of mortality rates. Column 2 pertains to the conversion (1-M5)^(1/5); Column 3 pertains to the conversion used in our study, viz., 1-(M5/5). (DOCX)

S6 Table Annual mortality rates (as percentages) implemented in Vortex, derived from West model life Table 5 [63]. (DOCX)

S7 Table Parameter settings for the basic Vortex model. In the third column, conservative estimates refer to estimates that can be expected to counteract the negative effects of inbreeding, Allee effects, and stochasticity. (DOCX)

S1 Fig Population growth factor (contour lines), for different values of the first year survival (*s*_*0*_) and the yearly adult reproduction rate (*m*). The red dashed line indicates combinations of *s*_*0*_ and *m* that yield a stable populations (i.e., populations with a growth factor of 1). Lower values for either of the two parameters result in extinction.
(DOCX)

S2 Fig Screenshot of Vortex’s graphical user interface. (DOCX)

S3 Fig Screenshot of the first part of a model input text-file. (DOCX)

S4 Fig Inbreeding and stochasticity: *I*_*risk*_ (lowest value of inbreeding depression parameter *I* that results in at least one extinction event in ten simulation runs) and *I*_*sure*_ (lowest value of inbreeding depression that results in extinction in all simulation runs) for various initial population sizes *N*_0_. (a) *f*_*i*_ = 30%; (b) *f*_*i*_ = 50%; and *f*_*i*_ = 70%. The horizontal dotted lines mark the range of values of *I* observed in AMHs.
(DOCX)
