# Supplementary material for: Inbreeding, Allee effects and stochasticity might be sufficient to account for Neanderthal extinction
Source: PLoS One. 2019 Nov 27;14(11):e0225117. doi: 10.1371/journal.pone.0225117 (PMC6880983; doi:10.1371/journal.pone.0225117)
Supplement: S1 Appendix — (DOCX) [file pone.0225117.s001.docx]

**S1 Appendix: Supplementary information**

Inbreeding, Allee effects and stochasticity might be sufficient to account for Neanderthal extinction

Krist Vaesen, Fulco Scherjon, Lia Hemerik, Alexander Verpoorte

**Description of the models**

**The deterministic matrix model**

The deterministic model consists of a Leslie matrix, a type of matrix that is commonly used in conservation biology to model population growth [58]. The matrix summarizes, for each of a population’s age classes, yearly survival and reproduction. Our model assumes a sex ratio of 1:1 and, because they are the limiting factor for reproduction, it only considers females.

*Calibration*

The basic matrix model, developed for purposes of calibration, did not involve inbreeding or Allee effects. To start, among extant hunter-gatherers, the age of first reproduction for females is, on average, 19-20 years, and the maximum age of reproduction for females is, on average, 33-34 years [62]. We therefore assumed that, in our modelled population, reproduction starts at the age of 19 years and ends at the age of 34 years. Correspondingly, classes 1-19 (0-1,..., 18-19y) comprise pre-reproductive females, classes 20-35 (19-20,..., 34-35) comprise reproductive females, and class 36 (females older than 35 years of age) comprise post-reproductive females. Because of the 36 classes, we arrive in Eqn (S1) at a matrix $C=(c_{i,j})$ with 36 rows (*i* =1,...,36) and 36 columns (*j* =1,...,36).

Eqn (S1)

The values on the subdiagonal in Eqn (S1) are the yearly survival probabilities. These were derived from West model life table 5 [63] and are presented in S1 Table. The only survival probability on the diagonal is the yearly survival probability for the post-reproductive females (i.e, $c_{36,36}$).

The top row in matrix *A* contains the yearly (non-zero) reproduction of the reproductive classes *i* = 20,..., 35. A given class’ yearly reproduction equals, following Caswell [58], the multiplication of the probability that the mother survives in the given year ($c_{i+1,i}$), the mean number of offspring per year (*m*=1/3, see [62,72], the fraction females in the offspring (1/2) and the probability to survive on average half a year for the offspring ($\sqrt{c_{2,1}}$).The square root in this last probability relates to the fact that survival is assumed to be homogeneously distributed over the year. If so, the probability to survive half a year times the probability to survive the next half year is the probability to survive one full year, in formula $\left(\sqrt{c_{2,1}}\right)^{2}=c_{2,1}$.

The solutions of the characteristic equation of matrix *C*, $\det\left(C-\lambda I\right)=0$, are the eigenvalues of the matrix. In this case the characteristic equation is as follows:

$\left(c_{36,36}-\lambda\right)\left(\lambda^{35}-\sum_{i=1}^{16} \lambda^{i-1}m\sqrt{c_{2,1}}\prod_{j=1}^{34-i} c_{j+1,j}\right)=0$ Eqn (S2)

From this, one can numerically derive the yearly population growth factor (this is the dominant eigenvalue, i.e. the eigenvalue *λ* of matrix *C* with the largest absolute value). The yearly growth factor (*λ*) is the number with which the population is multiplied per year; if it is less than 1, the population will go extinct. The relative growth rate is the natural logarithm of this number (ln(*λ*)). For our matrix parameterized with the values given in S1 Table we arrive at a dominant eigenvalue of 1.008, which corresponds to a relative growth rate (RGR) of 0.8% per year.

*Allee effects*

In Eqn (S3) we have modified the matrix to include the Allee effect *E* for the fraction of females breeding:

Eqn (S3)

The formula of *E* depends on the current number of individuals in the population *N*, the population’s carrying capacity *K*, the percentage of females breeding at low densities *p*_0_, the percentage of females breeding at carrying capacity *p_K_*, the Allee parameter *A* at low density, and the steepness parameter *B* at high density (i.e., close to carrying capacity *K*).

Eqn (S4)

In the most conservative scenario, in which reproduction at low and high densities are identical to reproduction without population density constraints (viz., 0.33), Eqn (S3) collapses into

Eqn (S5) (see also equation (1) in main text)

In order to assess the dependence of the population growth factor on the underlying parameters and how changes in these underlying parameters affect the growth factor one needs to determine the sensitivity of the growth factor to these parameters. The sensitivity and elasticity of the dominant eigenvalue to parameter *p* are defined as

and Eqn (S6)

The sensitivity *s*(*p*) represents the absolute change in the population growth factor due to an absolute change in the underlying parameter *p* $\partial\lambda/\partial p$, and the elasticity *e*(*p*) of an underlying parameter *p* represents the relative change in the population growth factor as a result of a relative change in the parameter *p$\left(p/\lambda\right)\cdot\left(\partial\lambda/\partial p\right)$.*

Our density-independent model comprises the parameters *m*, *s*_0_, *s*_1_, *s*_2_, *s*_3_, *s*_4_, *s*_5_, *s*_6_, *s*_7_ and *s*_8_. With the package Popbio in R [79] one can easily calculate the sensitivities and elasticities. S2 Table gives these values for the density-independent model (Eqn (S1)).

By gradually decreasing the yearly reproduction per female and holding all parameters at the values given in S1 Table, we can calculate the reproduction value for which the growth factor drops below 1 (and, thus, the population goes extinct). This happens when the reproduction value is below 0.26. When we instead decrease the first year survival and hold all parameters at the values given in S1 Table, the population goes extinct at first year survival values below 0.69. As can be seen in S1 Fig, the deterministic matrix model shows only population growth in the top right part of the figure. The contour line in red with value 1 for the dominant eigenvalue forms the border between growth and extinction.

S3 Table shows the results of the sensitivity analysis of the density-dependent model (i.e., the model that includes the Allee effect, or Eqn (S3)). *s*_2_, *s*_3_, *s*_4_, show the highest elasticity. Populations go extinct whenever *N*/(*N*+*A*)<0.79. Accordingly, S4 Table provides, per *N_0_*, the value, *A_sure(MM)_*, i.e., the lowest value for which the population goes extinct. It also gives, relying on Equation (1) in the main text, the corresponding birth interval (depicted in Fig 2b in the main text).

**The stochastic individual-based model**

For the IBM, we relied on VORTEX [59-61], a software package used by conservation biologists to perform Population Viability Analyses of endangered wildlife species. The tool allows modelling of extinction vortices that declining populations might undergo. VORTEX simulates the annual life events (e.g., sex determination, breeding, mortality) that might occur to each of the individuals within a given population, and records over time, in discrete time steps, the characteristics of these individuals as well as those of the population as a whole. Occurrences of events are probabilistic. Demographic stochasticity (relating to annual fluctuations in, e.g., sex ratios, births and deaths) is thus inherent to VORTEX models. The sequence of events in each time cycle is: Breed; Mortality; Age; Census. Extinction is reached when there are no males or females left in the population. Model parameters are entered by means of graphical user interface (see S2 Fig), or a model description text-file (see S3 Fig).

Our VORTEX models track, just like the matrix model, the size of Neanderthal populations over time. Since we wanted to avoid making assumptions about the superiority of AMHs over Neanderthals, all of the models were parameterized, as much as possible, based on estimates for AMHs.

*Calibration*

The basic IBM started, just like the matrix model, from the West model life table, Level 5 [63]. This table provides mortality rates for age cohorts of five years up till an age of 95 years. Vortex, in contrast, works with mortality rates for age cohorts of one year up till an age of 19 years. To implement the West model life table 5 in Vortex, we therefore did two things. First, mortality rates for the one-year age cohorts up till 19 years of age in Vortex were calculated by dividing the relevant West model life table 5’s five-year mortality rate by five (note that no such rescaling was necessary for the other parameters in the basic model, as they were available per year). In response to a comment by one of the reviewers of our study, we checked whether our rescaling procedure is accurate enough. The actual conversion from 5-year to 1-year rates should be 1-year probability = 5-year probability^(1/5)^. Now, S5 Table shows that for low mortality rates, our conversion and the latter conversion converge, and thus that our procedure is valid.

Second, the mortality rate for the >19 age cohort in Vortex was set at West model life table 5’s summed mortality rate for the 19-95 age cohort divided by 76 (i.e., the timespan between age 10 and age 95). S6 Table presents the resulting mortality rates of the basic Vortex model.

S7 Table provides an overview of and justification for the model’s other parameter settings. In case the value of a parameter was uncertain, we chose a conservative value, i.e., a value that can be expected to counteract (rather than to exacerbate) the negative effects of inbreeding, Allee effects and stochasticity. For instance, with respect to the population’s reproductive system, monogamy is a more conservative assumption than polygamy, as the latter plausibly increases the risk of inbreeding and makes mate-finding more problematic [80]. Therefore, the basic IBM assumes a monogamous mating system, even if it is unknown whether Neanderthals were in fact monogamous.

For various initial populations sizes (*N_0_* = 50; 100; 500; 1,000; 5,000), we ran ten simulation runs of the basic IBM, over a time span of 100 years. In order to compare the results with those obtained by means of the matrix model, we then calculated the relative growth rate (RGR), which is given by

RGR = (ln(*N*_100_)-ln(*N*_0_))/100 years Eqn (S7),

where *N_100_* is the size of the population after 100 years and *N_0_* is the initial population size. Averaged over all simulations, the average RGR was 0.76% per year.

*Inbreeding and stochasticity*

In VORTEX, inbreeding depression is modeled in terms of its effects on infant survival. It is governed by two parameters: the number of lethal equivalents, *I*, which is the number of recessive alleles carried in a heterozygous genome that would be lethal if carried in the homozygous state; and the percentage, *f_i_*, of the inbreeding depression caused by such lethal alleles rather than by other genetic mechanisms (e.g., a general disadvantage of homozygotes).

VORTEX models the effects of lethal alleles by, at the start of a simulation, assigning lethal alleles to individuals. When inbred individuals receive two copies of the same lethal allele, they are killed. To model the effects of inbreeding that are not due to lethal alleles, VORTEX applies an exponential equation to determine an individual’s reduction in survival.

In order to determine *I_risk_* and *I_sure_* for various initial population sizes (*N_0_* = 50; 100; 500; 1,000; 5,000), we introduced inbreeding into the basic model (see above), and varied *I* (*I*=20) from high to low. *I_risk_* is the lowest value of *I* that yields at least one extinction event in ten simulation runs, each run simulating over a time span of 10,000 years. *I_sure_* is the lowest value of *I* that yields extinction in all of the ten runs. We repeated this process for three values of *f_i_* (viz., *f_i_* = 30, 50, 70). Given that VORTEX models that incorporate inbreeding generally run very slow, and in particular when carrying capacity is high, we set *K* at 5,000 (or 10,000 in the basic model).

*Allee effects and stochasticity*

VORTEX uses Eqn (S4) to model Allee effects. In a conservative scenario, *p_0_* and *p_K_* equal the percentage of females breeding in the absence of population size constraints (viz., 0.33), and Eqn (S4) collapses into Eqn (S5). In this case, the Allee effect is a function merely of *N* and *A*. Accordingly, we needed only to simulate for different values of *A*. More specifically, we determined, for various initial population sizes (*N_0_* = 50; 100; 500; 1,000; 5,000), *A_risk_*, which is the lowest value of *A* that leads, in ten simulation runs with the same *A* value (each run comprises 10,000 years), to at least one extinction event. Also, we determined *A_sure_*, which is the lowest value of *A* for which all ten runs result in extinction.

*Inbreeding, Allee effects and stochasticity*

Estimates of *I* for modern humans [66-70] range from 0.58 [69] to 2.2 [66]. Gao et al. [69] point out that, since these estimates are based on reported deaths after birth and thus do not take into account prenatal deaths, the actual number of lethal equivalents might be higher; the authors surmise that prenatal deaths might increase *I* by one additional lethal equivalent (resulting in a maximum value of *I* = 3.2).

To assess the combined effects of inbreeding, Allee effect and stochasticity, we ran simulations for the highest *I*-value just reported (i.e., *I* = 3.2). More specifically, by varying widely varying over *A*, we determined for this *I*-value, and for various initial population sizes (*N_0_* = 50; 100; 500), *A_risk_*.

Although *I* = 3.2 is the least conservative value among the values found in the literature, our choice was motivated by the results obtained in scenarios that involved inbreeding alone (i.e., the results suggested that even at *I* = 3.2 the impact of inbreeding would be relatively small; see Results), and by the sluggishness of VORTEX in scenarios that combine Allee effects, inbreeding and very lengthy timespans (10,000 years). Also due to resource constraints, we did not determine *A_sure_*, restricted *N* to the range 0-500, and set carrying capacity at *K* = 5,000.

**The null models of Kolodny and Feldman**

Kolodny and Feldman [73] state that their models for the replacement of Neanderthals by AMHs may act as null models, in the sense that the models do not assume any selective advantage of AMHs over Neanderthals. Kolodny and Feldman’s basic, and most realistic, model starts from two demes: the first is located in Europe and the Levant, and initially comprises small groups of Neanderthals, the second is located in Africa, and is initially occupied by small bands of AMHs. Whenever, due to stochastic processes, a band in either of the demes dies out, it is replaced by a so-called propagule―i.e., a copy―of another, randomly selected, band. In case of the African deme, AMH bands will be replaced by a AMH propagule. In Europe and the Levant, in contrast, Neanderthal bands can be replaced by either a Neanderthal propagule or by a propagule of AMHs migrating that time step from Africa into Europe. Kolodny and Feldman thus assume, not unreasonably, that migration was largely uni-directional. The basic model now shows that under these conditions, even at low migration rates, the initial Neanderthal population would in the end be completely replaced by AMHs.

While it is correct that the model doesn’t assume any advantage of AMHs over Neanderthals when it comes to competition for spots that have become vacant in the European deme, it nonetheless does grant an advantage to AMHs. Note, first, that the number of bands in the African deme remains constant, since whenever such a band dies out, it is replaced by a *copy* of another AMH band. So the total number of AMH bands cannot decrease below the initial number of bands in the African deme. Further, migration in the model implies that an African AMH band can place a *copy* of itself in Europe. The growth factor relative to initial population size (i.e., population size at any given time over initial population size) of the total AMH population will thus always be ≥ 1. In contrast, the Neanderthal population cannot exceed its initial size, and will always have a growth factor relative to initial population size that is ≤ 1. Put differently, in Kolodny and Feldman’s basic model, AMHs have the advantage that they, in contrast to Neanderthals, can never go extinct.

The basic model, according to Kolodny and Feldman, is realistic in the sense that it involves only migration of AMHs from Africa into Europe and the Levant, and this, indeed, seems to correspond to what is observed in the archaeological record. Still, Kolodny and Feldman also consider scenarios in which migration is symmetric (i.e., as much migration of AMHs into Europe as migration of Neanderthals into Africa) and asymmetric (more migration of AMHs into Europe than migration of Neanderthals into Africa, as well as the reverse). The models for these scenarios do allow AMHs to disappear, but, apart from modelling implausible scenarios, they suggest that Neanderthals will disappear on the condition that the number of AMH migrants into Europe and the Levant is larger than the number of Neanderthal migrants into Africa. So, again, an advantage of AMHs over Neanderthals needs to be assumed for the latter to go extinct.

**References** *(references not included in main text)*

[79] Stubben CJ, Milligan BG. Estimating and analyzing demographic models using the popbio package in R. *J Statist Softw* 22: 11 (2007).

[80] Plesnar-Bielak A, Skrzynecka AM, Prokop ZM, Radwan J. Mating system affects population performance and extinction risk under environmental challenge. *Proc. R. Soc. B* 279: 4661–4466 (2012).

**TABLE CAPTIONS**

**S1 Table:** yearly survival probabilities based on the life tables published by [63]

**S2 Table:** Sensitivities and elasticities in the density-independent model (see third and fourth column)

**S3 Table:** Sensitivities and elasticities in the density-dependent model (see third and fourth column). Note that the Allee effect is included with specific parameter values

**S4 Table:** *A_sure(MM)_* and corresponding birth interval, for various initial population sizes *N_0_* (see Fig 2b in main text)

**S5 Table:** Comparison of 5y-to-1y conversion of mortality rates. Column 2 pertains to the conversion (1-M5)^(1/5); Column 3 pertains to the conversion used in our study, viz., 1-(M5/5)

**S6 Table:** Annual mortality rates (as percentages) implemented in Vortex, derived from West model life table 5 [63]

**S7 Table:** Parameter settings for the basic Vortex model. In the third column, conservative estimates refer to estimates that can be expected to counteract the negative effects of inbreeding, Allee effects, and stochasticity

**FIGURE CAPTIONS**

**S1 Fig:** Population growth factor (contour lines), for different values of the first year survival (*s_0_*) and the yearly adult reproduction rate (*m*). The red dashed line indicates combinations of *s_0_* and *m* that yield a stable populations (i.e., populations with a growth factor of 1). Lower values for either of the two parameters result in extinction.

**S2 Fig:** Screenshot of Vortex’s graphical user interface

**S3 Fig:** Screenshot of the first part of a model input text-file

**S4 Fig:** Inbreeding and stochasticity: *I_risk_* (lowest value of inbreeding depression parameter *I* that results in at least one extinction event in ten simulation runs) and *I_sure_* (lowest value of inbreeding depression that results in extinction in all simulation runs) for various initial population sizes *N*_0_. (a) *f_i_* = 30%; (b) *f_i_* = 50%; and *f_i_* = 70%. The horizontal dotted lines mark the range of values of *I* observed in AMHs.
