# Supplementary material for: Inbreeding, Allee effects and stochasticity might be sufficient to account for Neanderthal extinction
Source: PLoS One. 2019 Nov 27;14(11):e0225117. doi: 10.1371/journal.pone.0225117 (PMC6880983; doi:10.1371/journal.pone.0225117)
Supplement: S1 Table — (DOCX) [file pone.0225117.s002.docx]

|  | | | |
| --- | --- | --- | --- |
| Parameter(s) | Merged into | Description | Value |
| $c_{2,1}$ | $s_{0}$ | The probability to survive the first year | 0.8014 |
| $c_{3,2}$=$c_{4,3}$ =$c_{5,4}$ =$c_{6,5}$ | $s_{1}$ | The yearly probability to survive after the first year up to the fifth year | 0.9442 |
| $c_{7,6}$=$c_{8,7}$=$c_{9,8}$=$c_{10,9}$=$c_{11,10}$ | $s_{2}$ | The yearly probability to survive after the fifth year up to the tenth year | 0.9899 |
| $c_{12,11}$=$c_{13,12}$=$c_{14,13}$=$c_{15,14}$=$c_{16,15}$ | $s_{3}$ | The yearly probability to survive after the tenth year up to the fifteenth year | 0.9945 |
| $c_{17,16}$=$c_{18,17}$=$c_{19,18}$=$c_{20,19}$=$c_{21,20}$ | $s_{4}$ | The yearly probability to survive after the fifteenth year up to the twentieth year | 0.9924 |
| $c_{22,21}$=$c_{23,22}$=$c_{24,23}$=$c_{25,24}$=$c_{26,25}$ | $s_{5}$ | The yearly probability to survive after the twentieth year up to the twenty-fifth year | 0.9905 |
| $c_{27,26}$=$c_{28,27}$=$c_{29,28}$=$c_{30,29}$=$c_{31,30}$ | $s_{6}$ | The yearly probability to survive after the twenty-fifth year up to the thirtieth year | 0.9897 |
| $c_{32,31}$=$c_{33,32}$=$c_{34,33}$=$c_{35,34}$=$c_{36,35}$ | $s_{7}$ | The yearly probability to survive after the thirtieth year up to the thirty-fifth year | 0.9892 |
| $c_{36,36}$ | $s_{8}$ | The yearly probability to survive after the thirty-fifth year | 0.8729 |
