# Supplementary material for: Inbreeding, Allee effects and stochasticity might be sufficient to account for Neanderthal extinction
Source: PLoS One. 2019 Nov 27;14(11):e0225117. doi: 10.1371/journal.pone.0225117 (PMC6880983; doi:10.1371/journal.pone.0225117)
Supplement: S2 Table — (DOCX) [file pone.0225117.s003.docx]

| Parameter | Value | Sensitivity | Elasticity |
| --- | --- | --- | --- |
| *m* | 0.333 | 0.112 | 0.037 |
| *s*_0_ | 0.801 | 0.070 | 0.055 |
| *s*_1_ | 0.944 | 0.158 | 0.148 |
| *s*_2_ | 0.990 | 0.188 | 0.185 |
| *s*_3_ | 0.995 | 0.187 | 0.185 |
| *s*_4_ | 0.992 | 0.185 | 0.182 |
| *s*_5_ | 0.991 | 0.136 | 0.133 |
| *s*_6_ | 0.990 | 0.076 | 0.074 |
| *s*_7_ | 0.989 | 0.021 | 0.020 |
| *s*_8_ | 0.873 | 0.000 | 0.000 |
