# Supplementary material for: Inbreeding, Allee effects and stochasticity might be sufficient to account for Neanderthal extinction
Source: PLoS One. 2019 Nov 27;14(11):e0225117. doi: 10.1371/journal.pone.0225117 (PMC6880983; doi:10.1371/journal.pone.0225117)
Supplement: S3 Table — (DOCX) [file pone.0225117.s004.docx]

| Parameter | Value | Sensitivity | Elasticity |
| --- | --- | --- | --- |
| *s*_0_ | 0.801 | 0.072 | 0.056 |
| *s*_1_ | 0.944 | 0.163 | 0.150 |
| *s*_2_ | 0.989 | 0.195 | 0.187 |
| *s*_3_ | 0.995 | 0.194 | 0.187 |
| *s*_4_ | 0.992 | 0.191 | 0.184 |
| *s*_5_ | 0.991 | 0.135 | 0.130 |
| *s*_6_ | 0.990 | 0.071 | 0.068 |
| *s*_7_ | 0.990 | 0.019 | 0.018 |
| *s*_8_ | 0.873 | 0.000 | 0.000 |
| *N* | 500 | 0.000 | 0.001 |
| *K* | 4000 | 0.000 | 0.001 |
| *p_K_* | 30 | 0.001 | 0.037 |
| *p_0_* | 5 | 0.000 | 0.000 |
| *A* | 25 | 0.000 | -0.002 |
| *b* | 2 | 0.001 | 0.002 |
