# Supplementary material for: Inbreeding, Allee effects and stochasticity might be sufficient to account for Neanderthal extinction
Source: PLoS One. 2019 Nov 27;14(11):e0225117. doi: 10.1371/journal.pone.0225117 (PMC6880983; doi:10.1371/journal.pone.0225117)
Supplement: S4 Table — (DOCX) [file pone.0225117.s005.docx]

| ***N_0_*** | ***A_sure_MM_*** | ***Birth interval***  ***(years)*** |
| --- | --- | --- |
| 50 | 3 | 3.84 |
| 100 | 14 | 3.84 |
| 250 | 67 | 3.84 |
| 500 | 135 | 3.84 |
| 1,000 | 270 | 3.84 |
| 5,000 | 1,350 | 3.84 |
| 70,000 | 18,992 | 3.84 |
