# Supplementary material for: Inbreeding, Allee effects and stochasticity might be sufficient to account for Neanderthal extinction
Source: PLoS One. 2019 Nov 27;14(11):e0225117. doi: 10.1371/journal.pone.0225117 (PMC6880983; doi:10.1371/journal.pone.0225117)
Supplement: S5 Table — (DOCX) [file pone.0225117.s006.docx]

| **DATA** | **MATRIX** | **VORTEX** |
| --- | --- | --- |
| **5year mortality=M5** | **yearly survival**  **(1-M5)^(1/5)** | **yearly survival**  **1-(M5/5)** |
| 0.1 | 0.979 | 0.98 |
| 0.2 | 0.956 | 0.96 |
| 0.3 | 0.931 | 0.94 |
| 0.4 | 0.902 | 0.92 |
| 0.5 | 0.870 | 0.9 |
| 0.6 | 0.832 | 0.88 |
| 0.7 | 0.786 | 0.86 |
| 0.8 | 0.724 | 0.84 |
| 0.9 | 0.630 | 0.82 |
