# Supplementary material for: Inbreeding, Allee effects and stochasticity might be sufficient to account for Neanderthal extinction
Source: PLoS One. 2019 Nov 27;14(11):e0225117. doi: 10.1371/journal.pone.0225117 (PMC6880983; doi:10.1371/journal.pone.0225117)
Supplement: S6 Table — (DOCX) [file pone.0225117.s007.docx]

| **FEMALES** | | **MALES** | |
| --- | --- | --- | --- |
| **Age class** | **Mortality rate** | **Age class** | **Mortality rate** |
| Age 0 to 1 | 19.86 | Age 0 to 1 | 19.86 |
| Age 1 to 2 | 5.58 | Age 1 to 2 | 5.58 |
| Age 2 to 3 | 5.58 | Age 2 to 3 | 5.58 |
| Age 3 to 4 | 5.58 | Age 3 to 4 | 5.58 |
| Age 4 to 5 | 5.58 | Age 4 to 5 | 5.58 |
| Age 5 to 6 | 1.86 | Age 5 to 6 | 1.86 |
| Age 6 to 7 | 1.86 | Age 6 to 7 | 1.86 |
| Age 7 to 8 | 1.86 | Age 7 to 8 | 1.86 |
| Age 8 to 9 | 1.86 | Age 8 to 9 | 1.86 |
| Age 9 to 10 | 1.86 | Age 9 to 10 | 1.86 |
| Age 10 to 11 | 1.86 | Age 10 to 11 | 1.86 |
| Age 11 to 12 | 1.86 | Age 11 to 12 | 1.86 |
| Age 12 to 13 | 1.86 | Age 12 to 13 | 1.86 |
| Age 13 to 14 | 1.86 | Age 13 to 14 | 1.86 |
| Age 14 to 15 | 1.86 | Age 14 to 15 | 1.86 |
| Age 15 to 16 | 1.86 | Age 15 to 16 | 1.86 |
| Age 16 to 17 | 1.86 | Age 16 to 17 | 1.86 |
| Age 17 to 18 | 1.86 | Age 17 to 18 | 1.86 |
| Age 18 to 19 | 1.86 | Age 18 to 19 | 1.86 |
| Age >19 | 1 | Age >19 | 1 |
