# Supplementary material for: Inbreeding, Allee effects and stochasticity might be sufficient to account for Neanderthal extinction
Source: PLoS One. 2019 Nov 27;14(11):e0225117. doi: 10.1371/journal.pone.0225117 (PMC6880983; doi:10.1371/journal.pone.0225117)
Supplement: S7 Table — (DOCX) [file pone.0225117.s008.docx]

| **PARAMETER** | **INPUT** | **JUSTIFICATION** |
| --- | --- | --- |
| **Reproductive system** | | |
| Reproductive system | Monogamy, but with mate selection every year (serial monogamy) | Stable pairing during pregnancy and first couple of months after birth, but selection of new mate in the consecutive year.  Polygamy reduces effective population size, and thus increases risk of inbreeding [80]; plausibly, polygamy increases problems of mate-finding (Allee effects). Hence, monogamy is a more conservative assumption than polygamy. |
| Age of first offspring females | 19 | [62] |
| Maximum age of female reproduction | 34 | [62] |
| Age of first offspring males | 19 | [62] |
| Maximum age of male reproduction | 95 | Conservative estimate |
| Maximum lifespan | 95 | Conservative estimate |
| Maximum number of broods per year | 1 | Maximum number given a pregnancy of 6-9 + a weaning period of 3-4 months; conservative estimate |
| Maximum number of progeny per brood | 1 | Simplifying assumption |
| Sex ratio at birth (in % males) | 50% | Simplifying assumption |
| **Reproductive rates** | | |
| % adult females breeding each year | 33% | Average time between births is estimated at 3 years in extant hunter-gatherers [62] and Neanderthals [72] |
| % adult males in the pool of breeders | 100% | Conservative estimate |

*(continued next page)*

| **PARAMETER** | **INPUT** | **JUSTIFICATION** |
| --- | --- | --- |
| **Population parameters** | | |
| Initial population size (*N_0_*) | 50, 100, 250, 500, 1000, 5,000; for the matrix model, we also considered *N_0_* = 70,000 | range of census population size 5,000-70,000 [50] |
| Carrying capacity (*K*) | 10,000 | since our study is concerned primarily with what happens at low population numbers, we implemented a *K* that is considerably higher than the largest initial population size, i.e. *N_0_* = 5,000; by doing so, we avoid the dampening effects of carrying capacity; implementing even higher *K*-values makes VORTEX models too slow to run |
| Age distribution | proportional | See S6 Table |
