# Supplementary figures and images for: Inbreeding, Allee effects and stochasticity might be sufficient to account for Neanderthal extinction
Source: PLoS One. 2019 Nov 27;14(11):e0225117. doi: 10.1371/journal.pone.0225117 (PMC6880983; doi:10.1371/journal.pone.0225117)

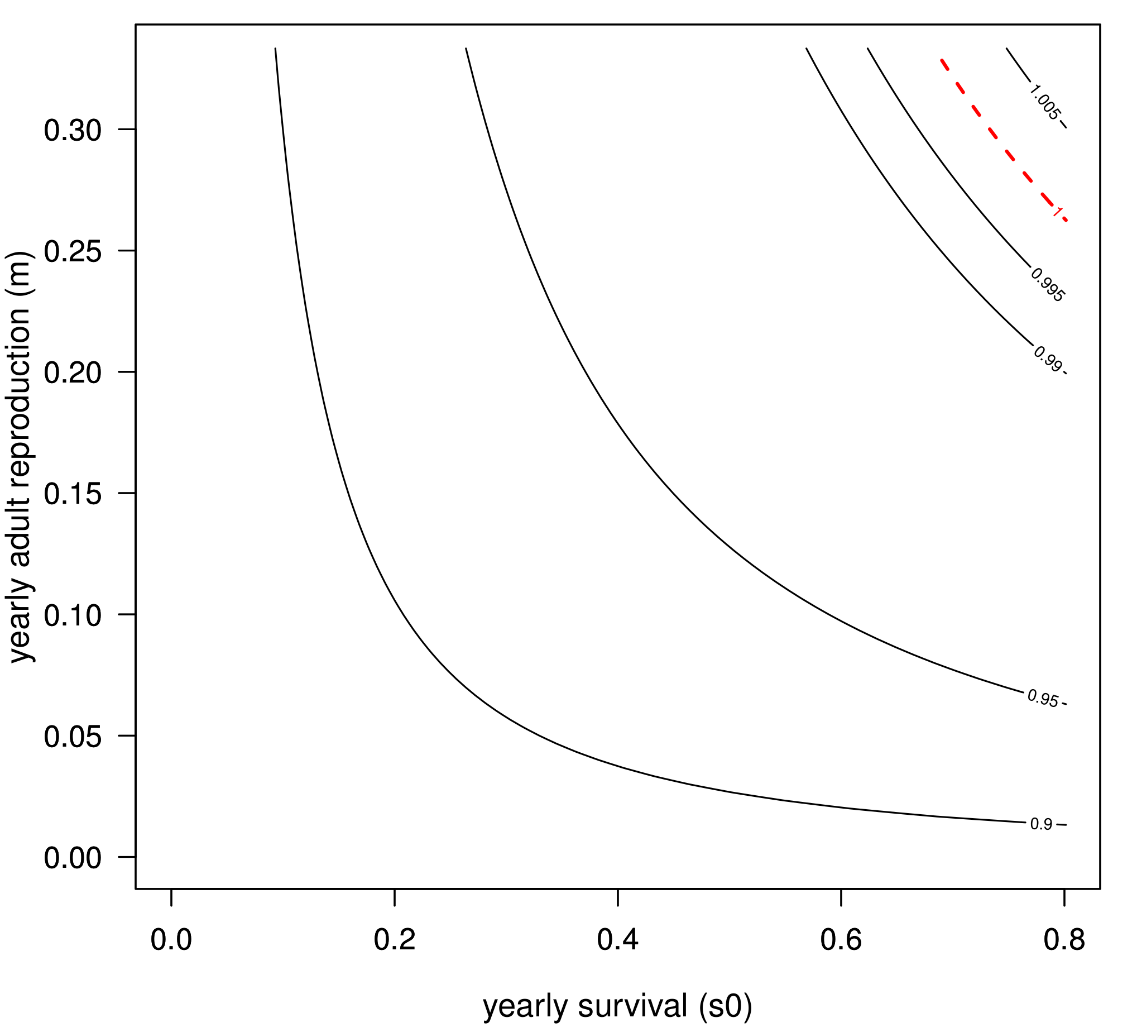

Supplement: S1 Fig — The red dashed line indicates combinations of s0 and m that yield a stable populations (i.e., populations with a growth factor of 1). Lower values for either of the two parameters result in extinction. (DOCX) [file pone.0225117.s009.docx]

**
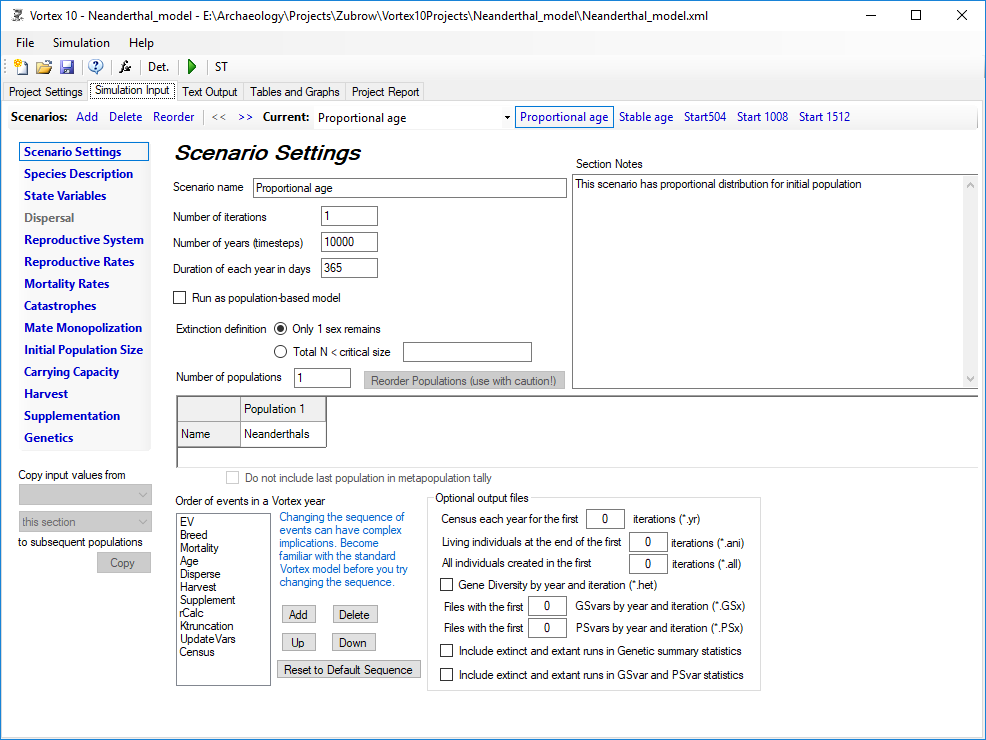
**

Supplement: S2 Fig — (DOCX) [file pone.0225117.s010.docx]

**
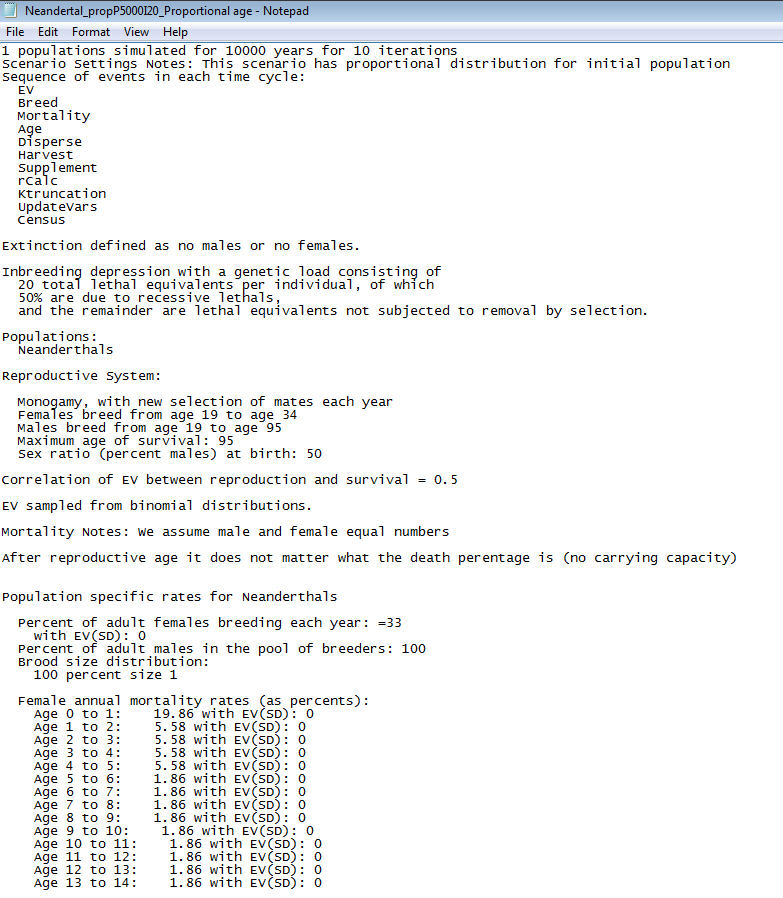
**

Supplement: S3 Fig — (DOCX) [file pone.0225117.s011.docx]

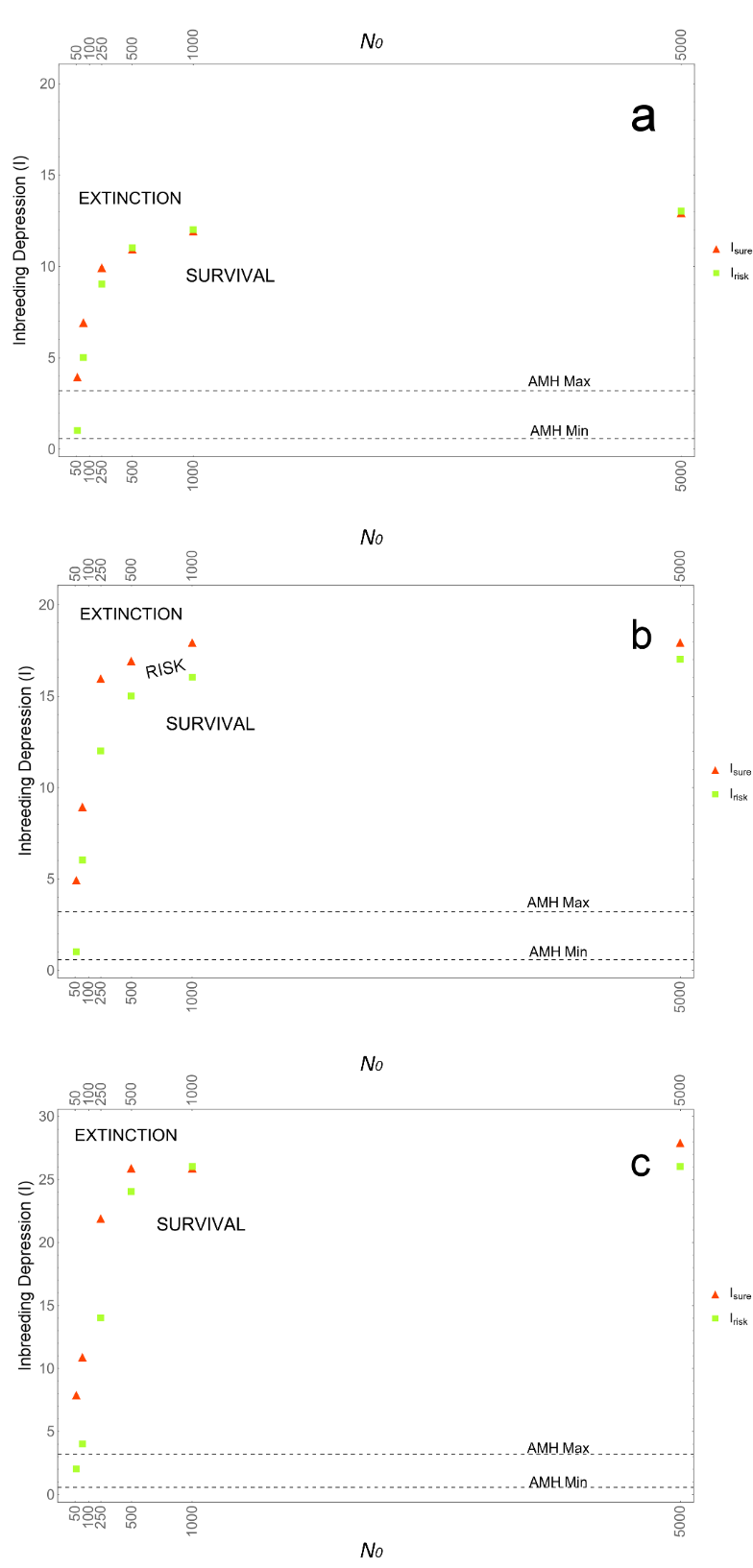

Supplement: S4 Fig — (a) fi = 30%; (b) fi = 50%; and fi = 70%. The horizontal dotted lines mark the range of values of I observed in AMHs. (DOCX) [file pone.0225117.s012.docx]
